# Trends and risk factors of patient falls in Korean hospitals: A five-year national reporting data analysis

**DOI:** 10.1371/journal.pone.0352198

**Published:** 2026-06-25

**Authors:** Jieun Shin, Yeon-Ju Baek, Nam-Yi Kim

**Affiliations:** 1 Department of Biomedical Informatics, College of Medicine, Konyang University, Daejeon, Republic of Korea; 2 Organ Transplantation Center, Konyang University Hospital, Daejeon, Republic of Korea; 3 Department of Nursing, Konyang University, Daejeon, Republic of Korea; Yonsei University Medical Center: Yonsei University Health System, KOREA, REPUBLIC OF

## Abstract

Patient falls are among the most frequent and preventable patient safety incidents in hospitals, often resulting in serious injury, prolonged hospitalization, and increased medical costs. Despite the nationwide implementation of the Korea Patient Safety Reporting and Learning System (KOPS), evidence on recent fall trends and risk factors across hospital types remains limited. This study analyzed five-year national reporting data to examine temporal trends and identify predictors of harmful inpatient falls in Korea. A retrospective cross-sectional analysis was conducted using 29,607 fall-related incidents reported to KOPS between 2020 and 2024. Variables included patient demographics, hospital characteristics, medical department, incident time, and level of harm, and multinomial logistic regression was used to identify factors associated with different levels of harm severity (near miss, adverse event, and sentinel event), with near miss as the reference category. Although the proportion of falls among all patient safety incidents declined from 56.0% in 2020 to 38.9% in 2024, the absolute number of reported falls continued to increase. Older adults aged 60 years or older and female patients had significantly higher odds of harmful falls. Long-term care hospitals and large hospitals with 500 beds or more showed elevated risks, while psychiatry and critical care departments recorded the highest proportions of sentinel events. These findings indicate that inpatient falls remain a major safety concern in Korea, particularly among older women and patients in large or long-term care hospitals. Differentiated, evidence-based prevention strategies—such as technology-assisted monitoring in large hospitals and improvements in workforce and environmental conditions in long-term care settings—are needed, alongside strengthened surveillance and data-driven safety management to reduce preventable harm.

## Introduction

Falls are among the most frequently reported and costly patient safety incidents in hospital settings worldwide. In acute care hospitals, the incidence of patient falls ranges from 3 to 11 per 1,000 patient days, and more than one-third of these cases result in physical injuries such as fractures or head trauma [1,2]. Falls not only pose a direct threat to patient safety but also lead to prolonged hospital stays, increased medical costs, functional decline, legal disputes, and reputational damage to healthcare institutions [1,3]. In particular, injurious falls are associated with high morbidity and mortality, highlighting the critical importance of early detection and prevention [1,2]. Consequently, the World Health Organization (WHO) identified the reduction of preventable harm, including falls, as a key priority in its *Global Patient Safety Action Plan 2021–2030* [4].

In South Korea, falls are among the most frequently reported types of patient safety incidents. Following the enactment of the *Patient Safety Act* in 2016, the Korean Patient Safety Reporting and Learning System (KOPS) was established as a nationwide reporting platform, and the mandatory reporting system for serious patient safety incidents was implemented in January 2021 [5,6]. The current hybrid reporting system—voluntary for most incidents and mandatory for serious events—aims to promote participation and improve data quality. However, persistent underreporting, variations in institutional reporting culture, and differences in patient populations across hospital types continue to limit the completeness and comparability of national fall data [7,8].

Previous studies have shown that older adults (particularly those aged 60 years and above) and female patients are at higher risk of falls, and that injurious falls occur more frequently in large tertiary hospitals that care for patients with higher acuity levels [8,9]. Hospital rooms, bathrooms, and hallways have been identified as high-risk locations, and the risk tends to increase during night shifts due to staff shortages and delayed response times [7,9]. Although pediatric falls are relatively rare, they remain clinically significant, with a hospital-based pediatric inpatient study indicating that approximately 16% of all falls result in injury [10]. International research has demonstrated that structured, patient-centered intervention programs—such as fall prevention toolkits—can effectively reduce both the frequency of falls and the rate of injurious falls [11].

Despite these findings, research in Korea remains limited. Most domestic studies have been confined to single institutions, specific patient groups, or short study periods, which limits the generalizability of their findings [8–10]. Moreover, few studies have utilized large-scale, nationally representative datasets that allow multi-year comparisons across diverse healthcare settings, and little is known about how fall characteristics differ by hospital type, bed capacity, or clinical department at the national level. Furthermore, the influence of Korea’s 2021 mandatory reporting policy on fall reporting patterns and harm severity has not been empirically evaluated, leaving an important gap in understanding national trends in fall-related safety incidents. To address these gaps, a long-term and comprehensive analysis based on nationwide reporting data is warranted. Indeed, the Second Comprehensive Patient Safety Plan (2023–2027) released by the Ministry of Health and Welfare identified falls and medication errors as the two most common types of patient safety incidents, emphasizing in-depth analysis and strengthened preventive measures as core strategies [3].

From an economic perspective, falls represent a substantial national burden. A pilot study in Korea estimated that preventable adverse events increased annual healthcare expenditures by approximately 870 billion KRW [5], while international studies have reported that patient safety incidents account for 1.8% to 16% of national healthcare spending [3,4]. Therefore, robust, reproducible evidence drawn from nationwide surveillance data is essential to support targeted prevention strategies and inform resource allocation within the healthcare system.

Accordingly, this study aimed to (1) identify trends in reported fall incidents in Korean hospitals over the past five years using national KOPS data; (2) determine demographic, institutional, and environmental risk factors associated with the severity of fall-related harm; and (3) explore the policy implications within Korea’s hybrid (voluntary and mandatory) reporting framework. The findings of this study are expected to provide empirical evidence to refine fall prevention strategies, strengthen hospital safety management, and improve the national patient safety reporting system.

## Methods

### Study design and data source

This retrospective cross-sectional study analyzed publicly available data from the Korea Patient Safety Reporting and Learning System (KOPS), which operates under the *Patient Safety Act*. KOPS collects voluntary reports of patient safety incidents from healthcare institutions, while mandatory reporting for serious patient safety incidents has been enforced since January 30, 2021.

In Korea, patient safety incidents are reported by designated patient safety officers, healthcare professionals, patients, or caregivers through the online KOPS platform (www.kops.or.kr) using a standardized reporting form. The form includes information on patient demographics, healthcare institution characteristics, incident details, and harm severity. Reported cases are reviewed and verified by staff at the Central Patient Safety Center, de-identified to remove personal identifiers, and then released as open data for research and policy use. Each year, KOPS aggregates data from the previous year and publicly discloses it on its website between May and August, allowing unrestricted access for analysis.

For this study, fall-related incident reports recorded between January 1, 2020, and December 31, 2024, were extracted from the KOPS database. All analyses were conducted using the most recently updated datasets available at the time of data retrieval, and only de-identified, publicly accessible data were used. Data were downloaded in comma-separated value (CSV) format directly from the KOPS Open Data portal to ensure reproducibility. The study data were accessed for research purposes on 21 November 2025. The authors did not have access to any information that could identify individual participants during or after data collection.

### Study population

**Fig 1:**
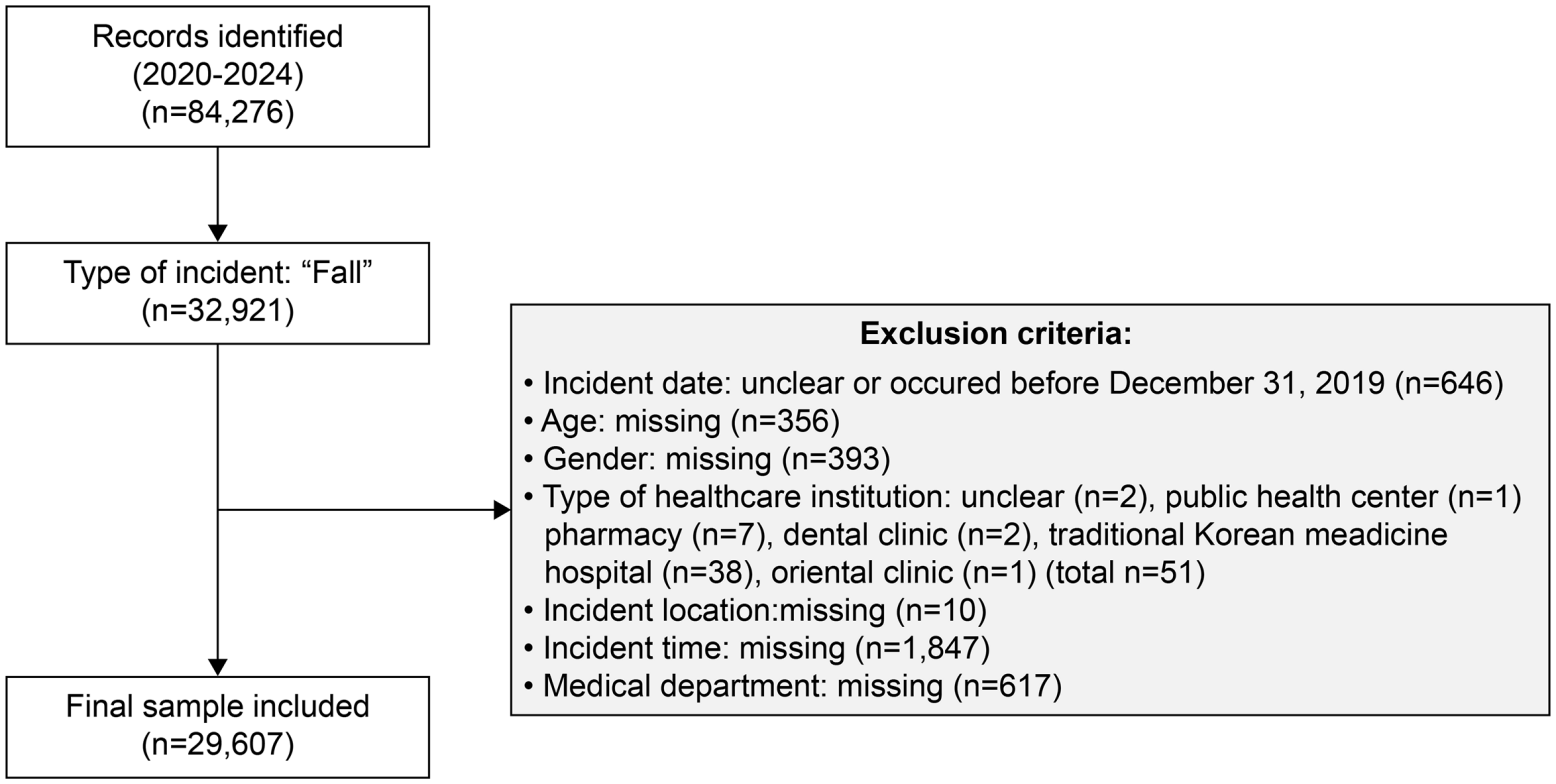
Flow diagram of study sample selection.

Among 84,276 patient safety incidents reported to KOPS between 2020 and 2024, a total of 32,921 cases classified as “falls” were identified. The KOPS open data used in this study were obtained from the KOPS Open Data portal as annual datasets (Patient Safety Incident Reports, 2020–2024), downloaded on November 21, 2025. The datasets were provided in CSV format. To ensure comparability across years, variables such as healthcare institution type and medical department were harmonized by recoding categories based on a unified classification scheme applied consistently across all study years. Reports with unclear or incomplete information were excluded, including those with (a) missing or pre-2019.12.31 occurrence dates (n = 646), (b) missing age (n = 356) or sex (n = 393), (c) unclear or non-hospital institution types such as public health centers, pharmacies, dental clinics, or oriental medical clinics (n = 51), (d) unspecified incident location (n = 10), (e) missing occurrence time (n = 1,847), or (f) missing treatment department information (n = 617). After applying these exclusion criteria, a total of 29,607 fall-related patient safety incidents were included in the final analysis (Fig 1). To ensure transparency and reproducibility, the number of excluded cases and the rationale for exclusion were documented a priori. No imputation was performed for missing data because the reporting system does not provide partial information for incomplete cases, and incomplete reports cannot be reliably interpreted.

### Variables and definitions

This study utilized fall-related patient safety incident data reported to the Korea Patient Safety Reporting and Learning System (KOPS). The original dataset includes a wide range of variables such as diagnosis, corrective actions after the incident, reporter information, and date of discovery. Based on previous studies [12] and patient safety agency guidelines [13], variables with potential relevance to the characteristics and severity of fall incidents were selected for analysis. The variables included gender, age, type of healthcare institution, bed capacity, medical department, incident time, and level of harm.

The level of harm from falls is originally classified in KOPS into six categories: *no harm*, *near miss*, *mild*, *moderate*, *severe*, and *death*. In this study, the severity of harm was reclassified into three categories to reflect both domestic hospital practice and international standards (Table 1).

**Table 1:** Classification of analysis variables.

Original Category (6 levels)	Grouped Category (3 groups)	Description
None Near miss	Near miss	Event that reached the patient but did not cause harm, or an error intercepted before reaching the patient
Mild harm Moderate harm	Adverse event	An incident that resulted in temporary or moderate patient harm
Severe harm Death	Sentinel event	A severe incident leading to serious harm or death

1. **Near miss** – includes cases classified as “no harm” or “near miss,” referring to events that did not reach the patient or reached the patient without causing harm.
2. **Adverse event** – includes cases classified as “mild” or “moderate,” defined as temporary or moderate harm requiring clinical intervention.
3. **Sentinel event** – includes cases classified as “severe” or “death,” indicating incidents that caused serious harm or resulted in patient death.

This three-tier classification aligns with widely adopted international patient safety frameworks and facilitates cross-study comparison and policy interpretation. Additional variable recoding was performed to standardize categorical values across reporting years (e.g., institution type, department classification), and all recoding procedures were documented to ensure reproducibility. No transformations were applied to continuous variables, as all variables in the dataset were originally categorical.

### Statistical analysis

All statistical analyses were performed using *IBM SPSS Statistics* version 29.0 (IBM Corp., Armonk, NY, USA).

Descriptive statistics, including frequencies and percentages, were used to summarize the characteristics of fall incidents. Differences in distributions across study years and harm severity categories were examined using the chi-square (χ²) test. Annual trends in the number and proportion of reported falls were presented descriptively, and temporal changes in fall rates were described through time-series comparisons.

To examine factors associated with fall severity, multinomial logistic regression analysis was conducted using a three-category outcome variable (near miss, adverse event, and sentinel event), with near miss as the reference category. Independent variables included gender, age group, type of healthcare institution, and time of occurrence. Adjusted odds ratios (aORs) and 95% confidence intervals (CIs) were calculated, and the results were visualized in Figure.

All statistical tests were two-tailed, and a p-value < 0.05 was considered statistically significant.

## Results

### General characteristics of fall incidents by year

As shown in Table 2, the total number of reported fall incidents showed a steady increase from 2020 to 2024. The gender distribution remained relatively stable over the five-year period, with males accounting for 46.6–48.7% and females for 51.3–53.4%, showing no statistically significant difference by year (χ² = 7.612, *p* = .107).

**Table 2:** Distribution of characteristics of patients with falls by year (2020–2024).

Variable	Categories	2020		2021		2022		2023		2024		*χ* ^*2*^	*p-value*
		n	(%)	n	(%)	n	(%)	n	(%)	n	(%)		
Gender	Male	2,698	(46.7)	2,645	(46.6)	2,522	(47.8)	3,026	(48.1)	3,206	(48.7)	7.612	0.107
	Female	3,074	(53.3)	3,030	(53.4)	2,759	(52.2)	3,265	(51.9)	3,382	(51.3)		
Age	0-19	89	(1.5)	77	(1.4)	119	(2.3)	141	(2.2)	127	(1.9)	42.298	< 0 .001
	20-59	1,020	(17.7)	1,096	(19.3)	991	(18.8)	1,070	(17.0)	1,087	(16.5)		
	≥ 60	4,663	(80.8)	4,502	(79.3)	4,171	(79.0)	5,080	(80.8)	5,374	(81.6)		
Type of healthcare institution	General hospital	3,038	(52.6)	3,213	(56.6)	3,120	(59.1)	3,549	(56.4)	3,611	(54.8)	343.400	< 0 .001
	Hospital	791	(13.7)	692	(12.2)	570	(10.8)	517	(8.2)	724	(11.0)		
	Long-term care hospital	1,890	(32.7)	1,572	(27.7)	1,360	(25.8)	1,914	(30.4)	1,926	(29.2)		
	others	53	(0.9)	198	(3.5)	231	(4.4)	311	(4.9)	327	(5.0)		
Bed capacity	< 500 beds	4076	70.6	3698	65.2	3285	62.2	3921	62.3	4591	69.7	197.440	< 0 .001
	≥500 beds	1685	29.2	1958	34.5	1946	36.8	2337	37.1	1957	29.7		
	No inpatient beds	11	0.2	19	0.3	50	0.9	33	0.5	40	0.6		
Medical department	Medical disciplines	2,290	(39.7)	2,296	(40.5)	2,124	(40.2)	2,513	(39.9)	2,516	(38.2)	146.040	< 0 .001
	Surgical disciplines	1,713	(29.7)	1,672	(29.5)	1,557	(29.5)	1,833	(29.1)	1,994	(30.3)		
	Diagnostic & support services	928	(16.1)	778	(13.7)	778	(14.7)	832	(13.2)	960	(14.6)		
	Emergency & critical care	64	(1.1)	99	(1.7)	95	(1.8)	116	(1.8)	59	(0.9)		
	Pediatrics	57	(1.0)	51	(0.9)	77	(1.5)	118	(1.9)	122	(1.9)		
	Psychiatry & mental health	255	(4.4)	362	(6.4)	335	(6.3)	392	(6.2)	439	(6.7)		
	Others	465	(8.1)	417	(7.3)	315	(6.0)	487	(7.7)	498	(7.6)		
Incident time	Day (07:00–14:59)	1,770	(31.0)	1,761	(31.4)	1,692	(32.2)	1,969	(31.5)	2,123	(32.4)	21.247	.047
	Evening (15:00–22:59)	1,744	(30.5)	1,728	(30.8)	1,633	(31.1)	1,956	(31.3)	1,948	(29.8)		
	Night (23:00–06:59)	2,204	(38.5)	2,128	(37.9)	1,922	(36.6)	2,331	(37.3)	2,472	(37.8)		
Level of harm	Near miss	1,817	(31.5)	2,240	(39.5)	1,872	(35.4)	2,266	(36.0)	2,373	(36.0)	97.642	< 0 .001
	Adverse event	3,914	(67.8)	3,392	(59.8)	3,364	(63.7)	3,942	(62.7)	4,154	(63.1)		
	Sentinel event	41	(0.7)	43	(0.8)	45	(0.9)	83	(1.3)	61	(0.9)		

In terms of age distribution, older adults (≥60 years) consistently represented the largest proportion of fall cases—80.8% in 2020, 79.3% in 2021, 79.0% in 2022, 80.8% in 2023, and 81.6% in 2024—with a statistically significant difference across years (χ² = 42.298, *p* < .001).

Regarding hospital size, the majority of falls occurred in small- and medium-sized hospitals (<500 beds), which accounted for 70.6% in 2020 and 69.7% in 2024. Large hospitals (≥500 beds) represented a smaller but stable proportion (29.2% in 2020 to 29.7% in 2024). The difference in fall distribution by bed capacity was statistically significant (χ² = 197.440, *p* < .001).

When comparing types of healthcare institutions, general hospitals consistently reported the highest proportion of falls, increasing from 52.6% in 2020 to 59.1% in 2022 before slightly decreasing to 54.8% in 2024. Variations among hospital, long-term care, and general hospitals were statistically significant (χ² = 343.400, *p* < .001).

By medical department, the majority of falls occurred in internal medicine wards (39.7–40.5%), followed by surgical wards (approximately 29–30%) and diagnostic support departments (approximately 13–16%), showing significant yearly differences (χ² = 146.040, *p* < .001).

Falls most frequently occurred during night shifts (36.6–38.5%), followed by daytime shifts (31.4–32.4%), with the evening shift accounting for 29–31%. The difference in fall occurrence by time of day was marginally significant (χ² = 21.247, *p* = .047).

Finally, with respect to the level of harm, the majority of falls were classified as *adverse events*, accounting for more than 60% of all cases (67.0% in 2020 and 63.1% in 2024). *Near misses* accounted for 31–36%, and *sentinel events* for approximately 1%. The difference in harm severity distribution by year was statistically significant (χ² = 97.642, *p* < .001).

### Trends of patient falls

**Fig 2:**
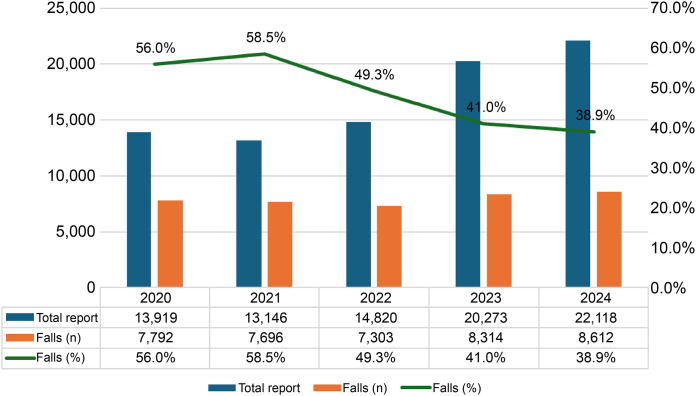
Annual trends in the number and proportion of fall incidents reported in the KOPS database, 2020–2024.

Between 2020 and 2024, the total number of patient safety incidents reported to the Korea Patient Safety Reporting and Learning System (KOPS) increased steadily from 13,919 in 2020–22,118 in 2024. In contrast, the proportion of incidents classified as patient falls decreased from 56.0% in 2020 to 38.9% in 2024 (Fig 2).

This downward trend indicates that although overall reporting activity has markedly expanded over the five-year period, the relative share of fall-related incidents declined as reporting of other event types—such as medication errors, pressure injuries, and diagnostic or communication-related incidents—became more active within the national reporting framework. The most notable decline occurred between 2021 and 2022, when the proportion of reported falls dropped from 58.5% to 49.3%, followed by a continued gradual decrease through 2024.

### Comparison of harm severity by patient characteristics

The distribution of harm severity (*near miss*, *adverse event*, *sentinel event*) according to patient characteristics is presented in Table 3. Statistically significant differences were observed across all variables (*p* < 0.001).

**Table 3:** Distribution of safety incident types by general characteristics of patients with falls.

Variable	Categories	Total		Near miss		Adverse event		Sentinel event		*χ* ^*2*^	*p-value*
		n	(%)	n	(%)	n	(%)	n	(%)		
Gender	Male	14097	(100.0)	5487	(38.9)	8462	(60.0)	148	(1.0)	131.2	< 0 .001
	Female	15510	(100.0)	5081	(32.8)	10304	(66.4)	125	(0.8)		
Age	0-19	553	(100.0)	284	(51.4)	269	(48.6)	0	(0.0)	157.83	< 0 .001
	20-59	5264	(100.0)	2163	(41.1)	3068	(58.3)	33	(0.6)		
	≥ 60	23790	(100.0)	8121	(34.1)	15429	(64.9)	240	(1.0)		
Type of healthcare institution	General hospital	16531	(100.0)	6845	(41.4)	9526	(57.6)	160	(1.0)	933.03	< 0 .001
	Hospital	3294	(100.0)	1407	(42.7)	1870	(56.8)	17	(0.5)		
	Long-term care hospital	8662	(100.0)	2052	(23.7)	6519	(75.3)	91	(1.1)		
	others	1120	(100.0)	264	(23.6)	851	(76.0)	5	(0.4)		
Bed capacity	< 500 beds	19571	(100.0)	6702	(34.2)	12704	(64.9)	165	(0.8)	61.053	< 0 .001
	≥500 beds	9883	(100.0)	3815	(38.6)	5962	(60.3)	106	(1.1)		
	No inpatient beds	153	(100.0)	51	(33.3)	100	(65.4)	2	(1.3)		
Medical department	Medical disciplines	11739	(100.0)	4205	(35.8)	7398	(63.0)	136	(1.2)	933.03	< 0 .001
	Surgical disciplines	8769	(100.0)	3462	(39.5)	5239	(59.7)	68	(0.8)		
	Diagnostic & support services	4276	(100.0)	1690	(39.5)	2560	(59.9)	26	(0.6)		
	Emergency & critical care	433	(100.0)	154	(35.6)	271	(62.6)	8	(1.8)		
	Pediatrics	425	(100.0)	169	(39.8)	256	(60.2)	0	(0.0)		
	Psychiatry & mental health	1783	(100.0)	390	(21.9)	1381	(77.5)	12	(0.7)		
	Others	2182	(100.0)	498	(22.8)	1661	(76.1)	23	(1.1)		
Incident time	Day (07:00–14:59)	9315	(100.0)	3211	(34.5)	5994	(64.3)	110	(1.2)	28.313	< 0 .001
	Evening (15:00–22:59)	9009	(100.0)	3313	(36.8)	5627	(62.5)	69	(0.8)		
	Night (23:00–06:59)	11057	(100.0)	3984	(36.0)	6981	(63.1)	92	(0.8)		

By gender, females had a higher proportion of *adverse events* (66.5%) compared with males (60.0%), whereas males showed a higher proportion of *near misses* (38.9% vs. 32.8%). The proportion of *sentinel events* was slightly higher in males (1.0%) than in females (0.8%) (χ² = 131.2, *p* < 0.001). According to age group, the highest proportion of *adverse events* occurred among patients aged ≥60 years (64.9%), followed by those aged 20–59 years (58.3%) and 0–19 years (48.6%). In the youngest age group (0–19 years), *near misses* accounted for more than half of all fall cases (51.4%), while *sentinel events* were most common in patients aged ≥60 years (1.0%) (χ² = 157.83, *p* < 0.001).

Regarding healthcare institution type, *adverse events* were most frequent in long-term care hospitals (75.3%), followed by general hospitals (57.6%) and hospitals (56.8%). Similarly, *sentinel events* were most frequent in long-term care hospitals (1.1%) (χ² = 933.0, *p* < 0.001).

By bed capacity, smaller hospitals (<500 beds) reported a higher proportion of *adverse events* (64.9%) compared with larger hospitals (≥500 beds, 60.3%). Conversely, *near misses* (38.6%) and *sentinel events* (1.1%) were slightly more frequent in large hospitals (χ² = 61.05, *p* < 0.001). In terms of medical department, *adverse events* were most frequent in psychiatry (77.5%) and other specialty units (76.1%), whereas *sentinel events* were highest in emergency and intensive care departments (1.8%) (χ² = 933.0, *p* < 0.001). Across incident times, *adverse events* occurred most frequently during the daytime (07:00–14:59, 64.3%), followed by the night (63.1%) and evening (62.5%) shifts. The highest proportion of *sentinel events* was also observed during the daytime (1.2%) (χ² = 28.31, *p* < 0.001).

From 2020 to 2024, significant differences were observed in harm severity according to hospital size, institution type, department, gender, and age group in all study years (*p* < 0.05). Detailed yearly distributions and subgroup comparisons are provided in Supplementary Table S1-S5 in S1 File.

### Risk factors associated with the severity of patient falls

**Fig 3:**
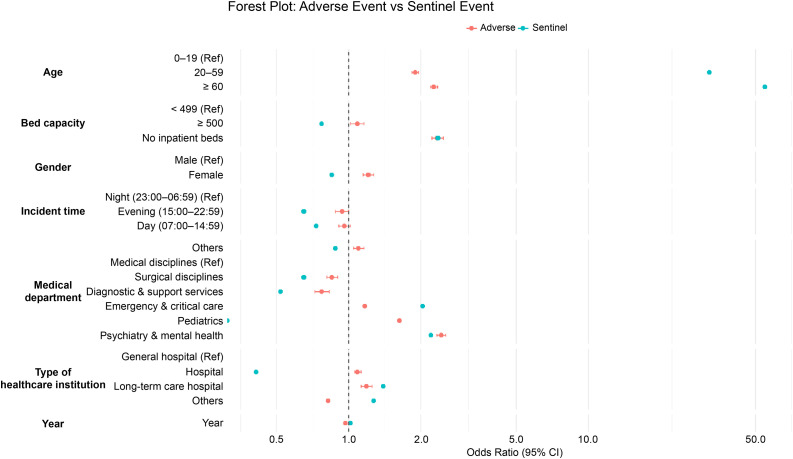
Adjusted odds ratios of risk factors for harmful falls.

The results of the multinomial logistic regression analysis, which examined factors associated with the occurrence of adverse events and sentinel events compared to near misses, are presented in Table 4 and Fig 3. Regarding gender, females were significantly more likely than males to experience an adverse event (OR = 1.21, 95% CI [1.15–1.27]). However, there was no statistically significant difference between genders regarding the likelihood of a sentinel event (OR = 0.85, 95% CI [0.66–1.08]). In terms of age groups, using those aged ≥60 years as the reference, the risk of adverse events was lower among both the 0–19 year group (OR = 0.44, 95% CI [0.34–0.57]) and the 20–59 year group (OR = 0.84, 95% CI [0.78–0.89]). For sentinel events, the 20–59 year group also showed a significantly lower risk compared to those aged ≥60 years (OR = 0.58, 95% CI [0.40–0.85]).

**Table 4:** Multinomial logistic regression analysis of factors associated with fall severity (reference: near miss).

Variable	Categories	Adverse event		Sentinel event	
		Adj. OR	95% CI	Adj. OR	95% CI
Gender	Male (ref)	–	–	–	–
	Female	1.21	[1.15-1.27]	0.85	[0.66-1.08]
Age	0-19	0.44	[0.34-0.57]		
	20-59	0.84	[0.78-0.89]	0.58	[0.4-0.85]
	≥ 60(ref)				
Bed capacity	< 500 beds (ref)	–	–	–	–
	≥500 beds	1.19	[1.12-1.26]	1.39	[1.02-1.88]
	No inpatient beds	0.81	[0.57-1.15]	1.28	[0.31-5.33]
Type of healthcare institution	General hospital (ref)	–	–	–	–
	Hospital	1.09	[1-1.19]	0.77	[0.44-1.35]
	Long-term care hospital	2.36	[2.19-2.53]	2.36	[1.67-3.33]
	Others	1.07	[0.85-1.33]	0.42	[0.13-1.34]
Medical department	Medical disciplines (ref)	–	–	–	–
	Surgical disciplines	0.85	[0.8-0.9]	0.65	[0.48-0.87]
	Diagnostic & support services	0.77	[0.71-0.84]	0.52	[0.33-0.82]
	Emergency & critical care	1.17	[0.95-1.44]	2.04	[0.97-4.26]
	Pediatrics	1.61	[1.19-2.17]		
	Psychiatry & mental health	2.45	[2.04-2.94]	2.20	[1.02-4.76]
	Others	1.10	[0.98-1.24]	0.88	[0.53-1.46]
Incident time	Day (07:00–14:59) (ref)	–	–	–	–
	Evening (15:00–22:59)	0.98	[0.92-1.04]	0.88	[0.64-1.21]
	Night (23:00–06:59)	1.04	[0.98-1.1]	1.37	[1.03-1.82]

As for hospital size, large hospitals with ≥500 beds were associated with a higher risk of both adverse events (OR = 1.19, 95% CI [1.12–1.26]) and sentinel events (OR = 1.39, 95% CI [1.02–1.88]) compared to smaller hospitals (<500 beds). According to institution type, when general hospitals were used as the reference, long-term care hospitals showed the highest likelihood of both adverse events (OR = 2.36, 95% CI [2.19–2.53]) and sentinel events (OR = 2.36, 95% CI [1.67–3.33]). By medical department, the likelihood of adverse events was highest in psychiatry (OR = 2.45, 95% CI [2.04–2.94]) and pediatrics (OR = 1.61, 95% CI [1.19–2.17]). Conversely, the risk was lower in surgical departments (OR = 0.85, 95% CI [0.80–0.90]) and diagnostic support departments (OR = 0.77, 95% CI [0.71–0.84]) compared to internal medicine. Regarding sentinel events, psychiatry (OR = 2.20, 95% CI [1.02–4.76]) presented a significantly higher risk, while surgical (OR = 0.65, 95% CI [0.48–0.87]) and diagnostic support departments (OR = 0.52, 95% CI [0.33–0.82]) showed relatively lower risks.

When comparing incident time using daytime (07:00–14:59) as the reference, falls occurring during the night shift (23:00–06:59) were significantly more likely to lead to sentinel events (OR = 1.37, 95% CI [1.03–1.82]). For adverse events, no significant differences were observed for either evening or night shifts compared to daytime. Overall, the risk of falls resulting in higher severity of harm was relatively higher among older adults (≥60 years), patients in long-term care hospitals, those in psychiatric departments, those in large hospitals (≥500 beds), and during night shifts.

## Discussion

This study analyzed 29,607 fall-related incidents reported to the Korea Patient Safety Reporting and Learning System (KOPS) from 2020 to 2024 to identify trends and risk factors associated with the severity of inpatient falls. Although the proportion of falls among all reported patient safety incidents decreased, the absolute number of fall cases continued to increase. The risk of injurious falls was particularly high among older adults (≥60 years), females, patients admitted to long-term care hospitals, and those in psychiatric departments. These findings are consistent with previous national and international studies [1,7,12].

The higher rate of falls in long-term care hospitals and chronic inpatient settings may be associated with differences in patient acuity, staffing levels, and environmental safety [9,10]. In psychiatric units, multiple factors—including psychotropic medication side effects, cognitive impairment, and behavioral instability— may contribute to the elevated fall risk [14]. The higher prevalence of falls among females may be explained by physiological vulnerabilities such as reduced muscle strength, osteoporosis, and impaired balance [15,16], highlighting the need for tailored fall prevention strategies targeting older female patients. However, no significant gender difference was observed in sentinel falls, suggesting that although women may experience falls more frequently, fall severity may be comparable between men and women.

One of the most notable findings was the higher occurrence of sentinel falls in both large hospitals (≥500 beds) and long-term care hospitals. This pattern reflects systemic differences in patient safety management by hospital type rather than purely environmental factors. Large hospitals typically treat a greater proportion of high-acuity patients and perform more complex medical procedures, which may be associated with a higher likelihood of severe harm following a fall [17]. Conversely, long-term care hospitals tend to serve frail, chronically ill patients with low staff-to-patient ratios, which may be associated with a higher risk of serious injury [18]. These differences underscore the need for institution-specific strategies rather than a uniform fall prevention approach.

The introduction of mandatory reporting for serious patient safety incidents in 2021 may have contributed to improvements in reporting accuracy; however, incomplete reporting and inter-institutional variability remain important challenges. Because KOPS includes both voluntary and mandatory reporting, trends in fall-related incidents may reflect changes in reporting behavior as well as true changes in event occurrence. Therefore, temporal changes should be interpreted with consideration of these system-level influences.

This study did not perform a formal causal evaluation of the 2021 policy, and the findings should be interpreted as descriptive. In addition, the estimates for sentinel events should be interpreted with caution, as their low frequency and the presence of zero cells in some subgroups (e.g., pediatrics) may lead to unstable estimates or complete separation in multinomial logistic regression models. Future studies should directly evaluate the impact of the 2021 mandatory reporting policy using pre–post or time-series analytical approaches.

The high rate of injurious falls in psychiatric wards further underscores the importance of a protective design for cognitive and mental health patients, as emphasized by the World Health Organization [13]. Given the growing burden of mental health conditions and the increased use of psychotropic medications in inpatient settings, more comprehensive safety interventions—combining environmental, clinical, and behavioral components—may be needed.

Collectively, these findings suggest that Korea’s patient safety management system is transitioning from quantitative expansion to qualitative improvement. Although the relative proportion of falls has decreased as reporting of other event types has expanded, falls still account for the largest share of all patient safety incidents. Preventive, evidence-based management systems remain essential. Consistent with the Second Comprehensive Patient Safety Plan (2023–2027) by the Ministry of Health and Welfare [3], this study provides empirical support for policies emphasizing fall prevention—especially among vulnerable populations such as the elderly and long-term care patients. From a public health perspective, reducing injurious falls is essential not only for improving patient safety but also for lowering long-term healthcare costs and mitigating functional decline in aging populations.

From a practical perspective, fall prevention strategies should be tailored to hospital size and patient population. Notably, falls occurred most frequently during night shifts, and night-time incidents were associated with significantly higher odds of sentinel events (OR 1.37). These findings emphasize the critical need for enhanced night-time surveillance and staffing protocols. Large tertiary hospitals, which manage complex and high-risk cases, should strengthen technological surveillance and real-time alert systems [19]. In contrast, long-term care hospitals should focus on workforce adequacy and environmental modifications to reduce fall hazards. The finding that falls most often occur during night shifts may reflect differences in patient monitoring, staffing patterns, or response times, and suggests the need to re-evaluate nurse staffing and patient monitoring protocols [20]. Such targeted approaches may be more effective than generalized interventions, particularly in resource-limited environments.

Internationally, the WHO [13] and the U.S. Agency for Healthcare Research and Quality [2] have demonstrated that multifactorial interventions—combining risk assessment, environmental design, and staff education—are among the most effective strategies for fall prevention. Aligning national strategies with these evidence-based approaches may enhance the effectiveness of fall prevention programs in Korea. In addition, integrating fall risk assessment tools into routine clinical workflows could improve early detection and reduce the likelihood of serious harm.

Despite its strengths, this study has several limitations. First, KOPS is a secondary, voluntary reporting system, which may result in variability in reporting accuracy and completeness across institutions. Therefore, the results should be interpreted as reflecting relative patterns rather than absolute incidence rates. Second, contextual details such as patient mobility status, medication use, or environmental conditions at the time of the fall were not available, limiting causal interpretation. Third, data on clinical outcomes such as length of stay, readmission, or cost burden were not included.

Nevertheless, this study provides valuable nationwide evidence based on large-scale patient safety reporting data. The standardized reporting structure of KOPS enhances comparability across institutions, but future improvements in reporting completeness and granularity would further strengthen surveillance efforts. Due to the nature of the reporting system, detailed contextual information—such as fall mechanism (e.g., bed-related, transfer-related, ambulation)—and patient-specific factors (e.g., comorbidities, medications, cognitive function) were unavailable. Future studies could address these gaps by linking KOPS data with electronic health records (EHRs), enabling more comprehensive risk prediction models that incorporate clinical and environmental factors.

Furthermore, integrating KOPS data with electronic health records (EHR) could enable the development of fall risk prediction models that incorporate clinical and contextual variables such as comorbid conditions, polypharmacy, and cognitive status. Recent advancements in artificial intelligence (AI) and machine learning may offer opportunities to enhance fall surveillance, automate early risk detection, and guide tailored interventions. The adoption of such data-driven safety management systems should be prioritized within Korean healthcare institutions.

## Conclusions

This study analyzed fall incidents reported to the Korea Patient Safety Reporting and Learning System (KOPS) from 2020 to 2024 to identify trends and risk factors associated with the severity of inpatient falls. While the proportion of falls among all reported patient safety incidents decreased, the absolute number of cases continued to rise. The risk of injurious falls was higher among older adults, females, patients admitted to long-term care hospitals or large hospitals (≥500 beds), and those in psychiatric or intensive care units.

These findings demonstrate that the characteristics and outcomes of falls vary according to hospital type and patient population. In large tertiary hospitals, complex medical procedures and a higher proportion of critically ill patients may increase the likelihood of serious fall-related harm, whereas in long-term care hospitals, cognitive impairment, chronic conditions, and limited staffing constitute major risk factors. Accordingly, institution-specific fall prevention strategies are essential: large hospitals should strengthen technological surveillance and real-time alert systems, while long-term care hospitals should focus on workforce reinforcement and environmental safety improvements.

By leveraging large-scale, nationally representative reporting data, this study offers evidence that can support targeted fall prevention policies and resource allocation in diverse healthcare settings. Continued refinement of national surveillance efforts, including improvements in reporting completeness and data quality, will further strengthen the ability to monitor fall trends and guide prevention strategies.

Future integration of KOPS data with electronic health records (EHRs) and the application of artificial intelligence–based prediction models may enhance the early identification of high-risk patients and support the development of more proactive, data-driven safety management systems aimed at reducing preventable harm.

## Supporting information

S1 File Supplementary Table S1–S5. Distribution of Safety Incident Types by General Characteristics of Patients with Falls in 2020–2024. This supplementary file contains five detailed tables presenting the distribution of patient safety incident types (near miss, adverse event, sentinel event) according to the general characteristics of patients with falls reported to the Korea Patient Safety Reporting and Learning System (KOPS) for each study year from 2020 to 2024. Supplementary Table S1. Distribution of Safety Incident Types by General Characteristics of Patients with Falls in 2020. Supplementary Table S2. Distribution of Safety Incident Types by General Characteristics of Patients with Falls in 2021. Supplementary Table S3. Distribution of Safety Incident Types by General Characteristics of Patients with Falls in 2022. Supplementary Table S4. Distribution of Safety Incident Types by General Characteristics of Patients with Falls in 2023. Supplementary Table S5. Distribution of Safety Incident Types by General Characteristics of Patients with Falls in 2024. Each table includes the frequency and percentage of near miss, adverse event, and sentinel event cases by key variables such as gender, age group, type of healthcare institution, bed capacity, medical department, and incident time. Chi-square (χ²) test results are provided to compare differences in harm severity across categories.
(DOCX)
