# Supplementary material for: Trends and risk factors of patient falls in Korean hospitals: A five-year national reporting data analysis
Source: PLoS One. 2026 Jun 25;21(6):e0352198. doi: 10.1371/journal.pone.0352198 (PMC13298754; doi:10.1371/journal.pone.0352198)
Supplement: S1 File — Distribution of Safety Incident Types by General Characteristics of Patients with Falls in 2020–2024. This supplementary file contains five detailed tables presenting the distribution of patient safety incident types (near miss, adverse event, sentinel event) according to the general characteristics of patients with falls reported to the Korea Patient Safety Reporting and Learning System (KOPS) for each study year from 2020 to 2024. Supplementary Table S1. Distribution of Safety Incident Types by General Characteristics of Patients with Falls in 2020. Supplementary Table S2. Distribution of Safety Incident Types by General Characteristics of Patients with Falls in 2021. Supplementary Table S3. Distribution of Safety Incident Types by General Characteristics of Patients with Falls in 2022. Supplementary Table S4. Distribution of Safety Incident Types by General Characteristics of Patients with Falls in 2023. Supplementary Table S5. Distribution of Safety Incident Types by General Characteristics of Patients with Falls in 2024. Each table includes the frequency and percentage of near miss, adverse event, and sentinel event cases by key variables such as gender, age group, type of healthcare institution, bed capacity, medical department, and incident time. Chi-square (χ²) test results are provided to compare differences in harm severity across categories. (DOCX) [file pone.0352198.s001.docx]

**Supplementary Tables**

**Supplementary Table S1. Distribution of Safety Incident Types by General Characteristics of Patients with Falls in 2020**

| **Variable** | **Categories** | **Total** | | **Near miss** | | **Adverse**  **event** | | **Sentinel event** | | ***χ^2^*** | ***p-value*** |
| --- | --- | --- | --- | --- | --- | --- | --- | --- | --- | --- | --- |
|  |  | **n** | **(%)** | **n** | **(%)** | **n** | **(%)** | **n** | **(%)** |  |  |
| Gender | Male | 2698 | (100.0) | 946 | (35.1) | 1729 | (64.1) | 23 | (0.9) | 32.476 | <0.001 |
|  | Female | 3074 | (100.0) | 871 | (28.3) | 2185 | (71.1) | 18 | (0.6) |  |  |
| Age | 0-19 | 89 | (100.0) | 51 | (57.3) | 38 | (42.7) | 0 | (0.0) | 64.612 | <0.001 |
|  | 20-59 | 1020 | (100.0) | 398 | (39.0) | 615 | (60.3) | 7 | (0.7) |  |  |
|  | ≥ 60 | 4663 | (100.0) | 1368 | (29.3) | 3261 | (69.9) | 34 | (0.7) |  |  |
| Type of healthcare institution | General hospital | 3038 | (100.0) | 1122 | (36.9) | 1885 | (62.0) | 31 | (1.0) | 265.43 | <0.001 |
|  | Hospital | 791 | (100.0) | 342 | (43.2) | 446 | (56.4) | 3 | (0.4) |  |  |
|  | Long-term care hospital | 1890 | (100.0) | 345 | (18.3) | 1538 | (81.4) | 7 | (0.4) |  |  |
|  | Others | 53 | (100.0) | 8 | (15.1) | 45 | (84.9) | 0 | (0.0) |  |  |
| Bed capacity | < 500 beds | 4591 | (100.0) | 1542 | (33.6) | 3010 | (65.6) | 39 | (0.8) | 44.023, | <0.001 |
|  | ≥500 beds | 1957 | (100.0) | 819 | (41.8) | 1117 | (57.1) | 21 | (1.1) |  |  |
|  | No inpatient beds | 40 | (100.0) | 12 | (30.0) | 27 | (67.5) | 1 | (2.5) |  |  |
| Medical department | Medical disciplines | 2290 | (100.0) | 769 | (33.6) | 1498 | (65.4) | 23 | (1.0) | 265.43 | <0.001 |
|  | Surgical disciplines | 1713 | (100.0) | 569 | (33.2) | 1134 | (66.2) | 10 | (0.6) |  |  |
|  | Diagnostic & support services | 928 | (100.0) | 324 | (34.9) | 602 | (64.9) | 2 | (0.2) |  |  |
|  | Emergency & critical care | 64 | (100.0) | 16 | (25.0) | 47 | (73.4) | 1 | (1.6) |  |  |
|  | Pediatrics | 57 | (100.0) | 22 | (38.6) | 35 | (61.4) | 0 | (0.0) |  |  |
|  | Psychiatry & mental health | 255 | (100.0) | 41 | (16.1) | 211 | (82.7) | 3 | (1.2) |  |  |
|  | Others | 465 | (100.0) | 76 | (16.3) | 387 | (83.2) | 2 | (0.4) |  |  |
| Incident time | Day (07:00-14:59) | 1770 | (100.0) | 542 | (30.6) | 1213 | (68.5) | 15 | (0.8) | 12.629 | 0.049 |
|  | Evening (15:00-22:59) | 1744 | (100.0) | 581 | (33.3) | 1149 | (65.9) | 14 | (0.8) |  |  |
|  | Night (23:00-06:59) | 2204 | (100.0) | 686 | (31.1) | 1506 | (68.3) | 12 | (0.5) |  |  |

**Supplementary Table S2. Distribution of Safety Incident Types by General Characteristics of Patients with Falls in 2021**

| **Variable** | **Categories** | **Total** | | **Near miss** | | **Adverse**  **event** | | **Sentinel event** | | ***χ^2^*** | ***p-value*** |
| --- | --- | --- | --- | --- | --- | --- | --- | --- | --- | --- | --- |
|  |  | **n** | **(%)** | **n** | **(%)** | **n** | **(%)** | **n** | **(%)** |  |  |
| Gender | Male | 2645 | (100.0) | 1099 | (41.6) | 1520 | (57.5) | 26 | (1.0) | 13.141 | 0.001 |
|  | female | 3030 | (100.0) | 1141 | (37.7) | 1872 | (61.8) | 17 | (0.6) |  |  |
| Age | 0-19 | 77 | (100.0) | 47 | (61.0) | 30 | (39.0) | 0 | (0.0) | 41.461 | <0.001 |
|  | 20-59 | 1096 | (100.0) | 502 | (45.8) | 589 | (53.7) | 5 | (0.5) |  |  |
|  | ≥ 60 | 4502 | (100.0) | 1691 | (37.6) | 2773 | (61.6) | 38 | (0.8) |  |  |
| Type of healthcare institution | General hospital | 3213 | (100.0) | 1474 | (45.9) | 1713 | (53.3) | 26 | (0.8) | 195.08 | <0.001 |
|  | Hospital | 692 | (100.0) | 302 | (43.6) | 386 | (55.8) | 4 | (0.6) |  |  |
|  | Long-term care hospital | 1572 | (100.0) | 419 | (26.7) | 1140 | (72.5) | 13 | (0.8) |  |  |
|  | others | 198 | (100.0) | 45 | (22.7) | 153 | (77.3) | 0 | (0.0) |  |  |
| Bed capacity | < 500 beds | 3698 | (100.0) | 1426 | (38.6) | 2248 | (60.8) | 24 | (0.6) | 11.786, | 0.019 |
|  | ≥500 beds | 1958 | (100.0) | 801 | (40.9) | 1138 | (58.1) | 19 | (1.0) |  |  |
|  | No inpatient beds | 19 | (100.0) | 13 | (68.4) | 6 | (31.6) | 0 | (0.0) |  |  |
| Medical department | Medical disciplines | 2296 | (100.0) | 871 | (37.9) | 1397 | (60.8) | 28 | (1.2) | 195.08 | <0.001 |
|  | Surgical disciplines | 1672 | (100.0) | 773 | (46.2) | 892 | (53.3) | 7 | (0.4) |  |  |
|  | Diagnostic & support services | 778 | (100.0) | 352 | (45.2) | 423 | (54.4) | 3 | (0.4) |  |  |
|  | Emergency & critical care | 99 | (100.0) | 37 | (37.4) | 61 | (61.6) | 1 | (1.0) |  |  |
|  | Pediatrics | 51 | (100.0) | 23 | (45.1) | 28 | (54.9) | 0 | (0.0) |  |  |
|  | Psychiatry & mental health | 362 | (100.0) | 72 | (19.9) | 289 | (79.8) | 1 | (0.3) |  |  |
|  | Others | 417 | (100.0) | 112 | (26.9) | 302 | (72.4) | 3 | (0.7) |  |  |
| Incident time | Day (07:00-14:59) | 1761 | (100.0) | 671 | (38.1) | 1069 | (60.7) | 21 | (1.2) | 10.137 | 0.119 |
|  | Evening (15:00-22:59) | 1728 | (100.0) | 680 | (39.4) | 1037 | (60.0) | 11 | (0.6) |  |  |
|  | Night (23:00-06:59) | 2128 | (100.0) | 867 | (40.7) | 1251 | (58.8) | 10 | (0.5) |  |  |

**Supplementary Table S3. Distribution of Safety Incident Types by General Characteristics of Patients with Falls in 2022**

| **Variable** | **Categories** | **Total** | | **Near miss** | | **Adverse**  **event** | | **Sentinel event** | | ***χ^2^*** | ***p-value*** |
| --- | --- | --- | --- | --- | --- | --- | --- | --- | --- | --- | --- |
|  |  | **n** | **(%)** | **n** | **(%)** | **n** | **(%)** | **n** | **(%)** |  |  |
| Gender | Male | 2522 | (100.0) | 999 | (39.6) | 1497 | (59.4) | 26 | (1.0) | 39.709 | <0.001 |
|  | female | 2759 | (100.0) | 873 | (31.6) | 1867 | (67.7) | 19 | (0.7) |  |  |
| Age | 0-19 | 119 | (100.0) | 54 | (45.4) | 65 | (54.6) | 0 | (0.0) | 26.082 | <0.001 |
|  | 20-59 | 991 | (100.0) | 404 | (40.8) | 584 | (58.9) | 3 | (0.3) |  |  |
|  | ≥ 60 | 4171 | (100.0) | 1414 | (33.9) | 2715 | (65.1) | 42 | (1.0) |  |  |
| Type of healthcare institution | General hospital | 3120 | (100.0) | 1237 | (39.6) | 1849 | (59.3) | 34 | (1.1) | 137.17 | <0.001 |
|  | Hospital | 570 | (100.0) | 241 | (42.3) | 326 | (57.2) | 3 | (0.5) |  |  |
|  | Long-term care hospital | 1360 | (100.0) | 312 | (22.9) | 1040 | (76.5) | 8 | (0.6) |  |  |
|  | others | 231 | (100.0) | 82 | (35.5) | 149 | (64.5) | 0 | (0.0) |  |  |
| Bed capacity | < 500 beds | 3285 | (100.0) | 1194 | (36.3) | 2070 | (63.0) | 21 | (0.6) | 9.7117, | 0.046 |
|  | ≥500 beds | 1946 | (100.0) | 665 | (34.2) | 1257 | (64.6) | 24 | (1.2) |  |  |
|  | No inpatient beds | 50 | (100.0) | 13 | (26.0) | 37 | (74.0) | 0 | (0.0) |  |  |
| Medical department | Medical disciplines | 2124 | (100.0) | 737 | (34.7) | 1362 | (64.1) | 25 | (1.2) | 137.17 | <0.001 |
|  | Surgical disciplines | 1557 | (100.0) | 602 | (38.7) | 945 | (60.7) | 10 | (0.6) |  |  |
|  | Diagnostic & support services | 778 | (100.0) | 322 | (41.4) | 451 | (58.0) | 5 | (0.6) |  |  |
|  | Emergency & critical care | 95 | (100.0) | 34 | (35.8) | 57 | (60.0) | 4 | (4.2) |  |  |
|  | Pediatrics | 77 | (100.0) | 28 | (36.4) | 49 | (63.6) | 0 | (0.0) |  |  |
|  | Psychiatry & mental health | 335 | (100.0) | 95 | (28.4) | 240 | (71.6) | 0 | (0.0) |  |  |
|  | Others | 315 | (100.0) | 54 | (17.1) | 260 | (82.5) | 1 | (0.3) |  |  |
| Incident time | Day (07:00-14:59) | 1692 | (100.0) | 591 | (34.9) | 1080 | (63.8) | 21 | (1.2) | 6.3129 | 0.389 |
|  | Evening (15:00-22:59) | 1633 | (100.0) | 590 | (36.1) | 1035 | (63.4) | 8 | (0.5) |  |  |
|  | Night (23:00-06:59) | 1922 | (100.0) | 678 | (35.3) | 1228 | (63.9) | 16 | (0.8) |  |  |

**Supplementary Table S4. Distribution of Safety Incident Types by General Characteristics of Patients with Falls in 2023**

| **Variable** | **Categories** | **Total** | | **Near miss** | | **Adverse**  **event** | | **Sentinel event** | | ***χ^2^*** | ***p-value*** |
| --- | --- | --- | --- | --- | --- | --- | --- | --- | --- | --- | --- |
|  |  | **n** | **(%)** | **n** | **(%)** | **n** | **(%)** | **n** | **(%)** |  |  |
| Gender | Male | 3026 | (100.0) | 1188 | (39.3) | 1799 | (59.5) | 39 | (1.3) | 26.619 | 0. <0.001 |
|  | female | 3265 | (100.0) | 1078 | (33.0) | 2143 | (65.6) | 44 | (1.3) |  |  |
| Age | 0-19 | 141 | (100.0) | 68 | (48.2) | 73 | (51.8) | 0 | (0.0) | 19.071 | 0.001 |
|  | 20-59 | 1070 | (100.0) | 421 | (39.3) | 639 | (59.7) | 10 | (0.9) |  |  |
|  | ≥ 60 | 5080 | (100.0) | 1777 | (35.0) | 3230 | (63.6) | 73 | (1.4) |  |  |
| Type of healthcare institution | General hospital | 3549 | (100.0) | 1515 | (42.7) | 1998 | (56.3) | 36 | (1.0) | 211.09 | <0.001 |
|  | Hospital | 517 | (100.0) | 209 | (40.4) | 305 | (59.0) | 3 | (0.6) |  |  |
|  | Long-term care hospital | 1914 | (100.0) | 469 | (24.5) | 1405 | (73.4) | 40 | (2.1) |  |  |
|  | others | 311 | (100.0) | 73 | (23.5) | 234 | (75.2) | 4 | (1.3) |  |  |
| Bed capacity | < 500 beds | 3921 | (100.0) | 1290 | (32.9) | 2575 | (65.7) | 56 | (1.4) | 45.29, | <0.001 |
|  | ≥500 beds | 2337 | (100.0) | 964 | (41.2) | 1347 | (57.6) | 26 | (1.1) |  |  |
|  | No inpatient beds | 33 | (100.0) | 12 | (36.4) | 20 | (60.6) | 1 | (3.0) |  |  |
| Medical department | Medical disciplines | 2513 | (100.0) | 926 | (36.8) | 1554 | (61.8) | 33 | (1.3) | 211.09 | <0.001 |
|  | Surgical disciplines | 1833 | (100.0) | 716 | (39.1) | 1091 | (59.5) | 26 | (1.4) |  |  |
|  | Diagnostic & support services | 832 | (100.0) | 316 | (38.0) | 507 | (60.9) | 9 | (1.1) |  |  |
|  | Emergency & critical care | 116 | (100.0) | 45 | (38.8) | 69 | (59.5) | 2 | (1.7) |  |  |
|  | Pediatrics | 118 | (100.0) | 48 | (40.7) | 70 | (59.3) | 0 | (0.0) |  |  |
|  | Psychiatry & mental health | 392 | (100.0) | 97 | (24.7) | 289 | (73.7) | 6 | (1.5) |  |  |
|  | Others | 487 | (100.0) | 118 | (24.2) | 362 | (74.3) | 7 | (1.4) |  |  |
| Incident time | Day (07:00-14:59) | 1969 | (100.0) | 688 | (34.9) | 1251 | (63.5) | 30 | (1.5) | 10.619 | 0.101 |
|  | Evening (15:00-22:59) | 1956 | (100.0) | 722 | (36.9) | 1215 | (62.1) | 19 | (1.0) |  |  |
|  | Night (23:00-06:59) | 2331 | (100.0) | 850 | (36.5) | 1447 | (62.1) | 34 | (1.5) |  |  |

**Supplementary Table S5. Distribution of Safety Incident Types by General Characteristics of Patients with Falls in 2024**

| **Variable** | **Categories** | **Total** | | **Near miss** | | **Adverse**  **event** | | **Sentinel event** | | **χ^2^** | **p-value** |
| --- | --- | --- | --- | --- | --- | --- | --- | --- | --- | --- | --- |
|  |  | **n** | **(%)** | **n** | **(%)** | **n** | **(%)** | **n** | **(%)** |  |  |
| Gender | Male | 3206 | (100.0) | 1255 | (39.1) | 1917 | (59.8) | 34 | (1.1) | 28.682 | <0.001 |
|  | female | 3382 | (100.0) | 1118 | (33.1) | 2237 | (66.1) | 27 | (0.8) |  |  |
| Age | 0-19 | 127 | (100.0) | 64 | (50.4) | 63 | (49.6) | 0 | (0.0) | 24.406 | <0.001 |
|  | 20-59 | 1087 | (100.0) | 438 | (40.3) | 641 | (59.0) | 8 | (0.7) |  |  |
|  | ≥ 60 | 5374 | (100.0) | 1871 | (34.8) | 3450 | (64.2) | 53 | (1.0) |  |  |
| Type of healthcare institution | General hospital | 3611 | (100.0) | 1497 | (41.5) | 2081 | (57.6) | 33 | (0.9) | 195.84 | <0.001 |
|  | Hospital | 724 | (100.0) | 313 | (43.2) | 407 | (56.2) | 4 | (0.6) |  |  |
|  | Long-term care hospital | 1926 | (100.0) | 507 | (26.3) | 1396 | (72.5) | 23 | (1.2) |  |  |
|  | others | 327 | (100.0) | 56 | (17.1) | 270 | (82.6) | 1 | (0.3) |  |  |
| Bed capacity | < 500 beds | 4591 | (100.0) | 1542 | (33.6) | 3010 | (65.6) | 39 | (0.8) | 44.023, | <0.001 |
|  | ≥500 beds | 1957 | (100.0) | 819 | (41.8) | 1117 | (57.1) | 21 | (1.1) |  |  |
|  | No inpatient beds | 40 | (100.0) | 12 | (30.0) | 27 | (67.5) | 1 | (2.5) |  |  |
| Medical department | Medical disciplines | 2516 | (100.0) | 902 | (35.9) | 1587 | (63.1) | 27 | (1.1) | 195.84 | <0.001 |
|  | Surgical disciplines | 1994 | (100.0) | 802 | (40.2) | 1177 | (59.0) | 15 | (0.8) |  |  |
|  | Diagnostic & support services | 960 | (100.0) | 376 | (39.2) | 577 | (60.1) | 7 | (0.7) |  |  |
|  | Emergency & critical care | 59 | (100.0) | 22 | (37.3) | 37 | (62.7) | 0 | (0.0) |  |  |
|  | Pediatrics | 122 | (100.0) | 48 | (39.3) | 74 | (60.7) | 0 | (0.0) |  |  |
|  | Psychiatry & mental health | 439 | (100.0) | 85 | (19.4) | 352 | (80.2) | 2 | (0.5) |  |  |
|  | Others | 498 | (100.0) | 138 | (27.7) | 350 | (70.3) | 10 | (2.0) |  |  |
| Incident time | Day (07:00-14:59) | 2123 | (100.0) | 719 | (33.9) | 1381 | (65.0) | 23 | (1.1) | 11.774 | 0.067 |
|  | Evening (15:00-22:59) | 1948 | (100.0) | 740 | (38.0) | 1191 | (61.1) | 17 | (0.9) |  |  |
|  | Night (23:00-06:59) | 2472 | (100.0) | 903 | (36.5) | 1549 | (62.7) | 20 | (0.8) |  |  |
